# When AI Colludes: Clinical Reliability of Training and Preference Data as a Trustworthy-AI Criterion

**DOI:** 10.2196/96894

**Published:** 2026-05-26

**Authors:** Hina Tahseen

**Affiliations:** 1 Somerset NHS Foundation Trust, Summerlands Hospital Site, Yeovil, England, BA202BX, United Kingdom, 44 01935410784; 2 School of Medicine, Cardiff University, Cardiff, Wales, United Kingdom

**Keywords:** artificial intelligence, cognitive bias, training data, collusion, sycophancy, large language models, mental health, AI safety, reinforcement learning from human feedback, AI governance

## Abstract

Research on artificial intelligence (AI) and mental health has focused largely on harms at deployment, including chatbot safety, sycophancy, and AI-associated delusions. Less attention has been paid to a prior question: whether the human-generated text and preference judgments that shape large language models are themselves clinically reliable, particularly when self-report may be distorted. This Viewpoint aims to develop the clinical psychiatric construct of collusion—the uncritical acceptance of an unreliable account—as an analytic lens for AI training and deployment, and to argue that the clinical reliability of training and preference data should be treated as an explicit trustworthy-AI criterion in mental-health–relevant systems. A conceptual synthesis of psychiatry, clinical psychology, and AI safety literature was undertaken. The analysis distinguishes three pipeline layers: pretraining corpora, preference data and posttraining methods, and deployment-time interaction. It maps the clinical construct of collusion against adjacent technical concepts, including sycophancy, reward overoptimization, grounding, refusal training, red-teaming, and live monitoring. The synthesis suggests that collusion-like dynamics are least applicable at the pretraining layer and most applicable at the preference-data and deployment layers, where unassessed user or labeler input can be reinforced without corroboration. Existing mitigations, including data curation, Constitutional AI, reward-model evaluation, grounded generation, refusal training, red-teaming, and postdeployment monitoring, address parts of this problem. However, these approaches are not yet organized around a clinically informed account of when self-report is unreliable. The central novelty is therefore not a generic claim about bias, but the proposal that clinical self-report reliability should be assessed as a distinct data-quality and governance dimension. Trustworthy-AI frameworks for mental-health–relevant applications should incorporate clinical expertise in self-report reliability into preference-data design, red-teaming, and postmarket surveillance. Adding the clinical reliability of training and preference data as an explicit criterion could complement existing technical safeguards while leaving empirical evaluation of clinician involvement as an open research agenda.

## Introduction

Research on artificial intelligence (AI) and mental health has expanded rapidly. Systematic reviews have evaluated the capabilities and limitations of generative AI in mental health applications [1]. Simulation work has shown that AI chatbots frequently fail to challenge delusional content and may exhibit sycophantic behavior that reinforces harmful beliefs [2,3]. Clinical case series and rapid scoping reviews of media reporting have documented AI-associated delusions and adverse psychiatric events in users of large language models (LLMs), including individuals with no prior psychotic history [4,5]. Large-scale analyses of deployed assistants have begun to quantify the prevalence of potentially disempowering interactions [6].

This literature concentrates on what happens at the point of interaction between a user and a deployed system. A logically prior question has received less attention: whether the human-generated text and human preference judgments that shape these systems are themselves clinically reliable. The technical literature is not silent on related phenomena. Sycophancy, reward overoptimization, specification gaming, and disempowerment potential are well-developed constructs in AI research [6-9]. Fairness-aware machine learning and participatory and community-engaged approaches to data curation have addressed demographic representation, population-level bias, and inclusion in training corpora [10,11]. Adjacent work on data integrity in materials science similarly shows how flawed and biased scientific data can be amplified in AI systems, but addresses domain-level data integrity rather than clinical self-report reliability [12]. These contributions are substantial and are not replicated here. The contribution this Viewpoint advances is narrower and complementary: that psychiatry and clinical psychology have a mature vocabulary and a working evidence base for assessing when self-report is unreliable, and that this expertise is currently absent from the curation of training corpora, the design of preference data, and the evaluation of trustworthy AI in health care. The distinction is important. Existing data-quality work asks who is represented in training corpora and how proportionally. The question raised here is different: whether the human-generated text those populations produced is a reliable account of experience, cognition, and need in the specific sense developed in the clinical self-report literature. This is not a question of demographic representation but of self-report reliability, and it is a question that clinical disciplines are specifically trained to assess.

To make that case rigorously, *collusion* is developed below as an analytic construct rather than a metaphor, with stated necessary conditions, explicit disanalogies with the clinical encounter, and a mapping against adjacent technical constructs.

## A Three-Layer Account of the Pipeline

A common limitation of clinically motivated commentary on AI is to treat “training data” as a single object. The argument is sharper when three pipeline layers are distinguished.

### Pretraining Corpora

General-purpose LLMs are first trained on large heterogeneous text corpora drawn from web content, books, code, and other sources. Recent work shows that data selection, filtering, deduplication, and source mixing materially affect model performance and downstream behavior, and that pretraining corpora are an active object of research and curation [13,14]. These corpora are not equivalent to validated clinical truth, but they also contain corrective material such as textbooks and peer-reviewed literature; the relevant claim is selection-weighted bias rather than absence of accurate text.

### Preference Data and Posttraining

Models are then refined through reinforcement learning from human feedback, direct preference optimization, or rule-based variants such as Constitutional AI and reinforcement learning from AI feedback [15]. Recent technical work has shown that human preference judgments can favor view-matching or agreeable responses over truthful ones, that reward models trained on such judgments can be overoptimized, and that dedicated evaluation of reward models is required [7,16,17].

### Deployment-Time Interaction

Final behavior is further shaped by system prompts, interface incentives, retrieved context, memory, personalization, and the accumulation of multiturn interaction history. Sycophancy has been shown to increase with extended interaction and personalization [9], and providers have publicly described episodes in which deployment-time tuning and feedback design induced or worsened sycophantic behavior in production systems [18].

The collusion analogy is weakest at the pretraining layer, where the relevant pathology is corpus composition rather than reinforced agreement, and is strongest at the preference-data and deployment layers, where systems are explicitly optimized to produce more of what users or labelers approve.

## Cognitive Distortion and the Clinical Reliability of Self-Report

The cognitive science underpinning the argument is well established but should not be overstated. Tversky and Kahneman [19] demonstrated that human reasoning shows systematic deviations from normative models under uncertainty, and Kahneman’s later synthesis treats heuristic processing as a default operating mode of cognition [20]. The strength of this view is contested by the ecological-rationality tradition, which argues that heuristics are often well calibrated to environmental structure [21]. The conservative claim sufficient for the present argument is that human-generated text reflects systematic, predictable cognitive biases that pretraining corpora carry forward without correction.

Clinical populations introduce a further layer of distortion that the cognitive bias literature alone does not capture. Beck’s cognitive model, developed and validated as a framework for psychotherapy rather than as a general theory of cognition, describes recurrent patterns of distorted thinking in mood and anxiety disorders, including catastrophizing, overgeneralization, dichotomous reasoning, and selective abstraction [22]. Psychotic disorders disrupt the cognitive architecture on which accurate self-report depends; ambulatory and self-report studies in psychosis have documented the methodological challenges of obtaining valid first-person data even under controlled conditions [23]. Severe depression is characterized by psychomotor retardation, anhedonia, and social withdrawal, and the digital phenotyping literature has reported associations between depressive symptoms and reduced or altered patterns of digital and social media engagement [24]; affected individuals are therefore plausibly underrepresented, or differently represented, in pretraining corpora drawn from public text. These illustrations are clinical hypotheses about selection and presentation effects in pretraining corpora; they have not been quantified in any specific corpus and are offered as examples, not as epidemiologically calibrated estimates.

What clinical practice routinely contributes is the assessment of reliability in self-report: holding an account against observation, collateral history, illness pattern, secondary gain, and the pragmatic context of the encounter. A patient facing detention under the Mental Health Act may minimize psychotic symptoms to preserve autonomy. A patient seeking controlled medication may exaggerate distress. A patient whose life has been shaped by years of institutional care may have internalized a framework for understanding their own needs that bears little resemblance to standardized assessment instruments. The clinician’s task is not to disbelieve, but to test the account.

## Collusion as an Analytic Construct

In clinical usage, *collusion* denotes the uncritical acceptance by a clinician of a patient account that is unreliable in ways the patient may not recognize, in the absence of corroboration against observation, collateral history, or known illness patterns. It is treated as a clinical error, regardless of whether the patient was being dishonest.

Adapted to AI systems, collusion can be defined as follows: *the structural reinforcement of user input as ground truth in the absence of mechanisms (at training, preference labeling, or deployment) for assessing the clinical reliability of that input*. On this definition, the necessary conditions for collusion-like dynamics in an AI system are: (1) input from a source whose reliability is unassessed, (2) optimization pressure that rewards agreement with that input, and (3) the absence of any corroboration mechanism. Under current preference-optimization regimes, these conditions are routinely met at the preference-data and deployment layers.

Several disanalogies must be stated explicitly. AI systems have no intent, no dyadic relational dynamic, and no countertransference; the “patient” role is distributed across millions of unidentified users, the “clinician” role is distributed across data curators and preference labelers, and there is no professional duty to a specific person. The analogy is therefore structural rather than psychodynamic.

Several adjacent constructs in the technical literature describe overlapping phenomena. *Sycophancy* names the behavioral pattern of agreeing with users against the system’s own evidence [7,9]. *Reward hacking* and *reward overoptimization* name the optimization pathology in which a model exploits an imperfect reward signal [16,17]. *Specification gaming* generalizes this to misaligned objectives. *Epistemic deference* and *perspective mimesis* describe deployment-time effects on user belief [9]. *Disempowerment potential* names the empirical correlate at scale [6]. The contribution of *collusion* is not to compete with these constructs but to name what they do not foreground: the *clinical reliability of the input* on which the system is being optimized. Collusion is offered as a clinically informed redescription of a family of already described technical phenomena, not as a claim that the field has ignored bias or sycophancy.

## The Feedback Loop in Deployed Systems

The empirical correlate at deployment scale is informative but should be reported precisely. Anthropic- and University of Toronto–affiliated researchers analyzed approximately 1.5 million Claude.ai conversations and developed a framework for assessing disempowerment potential across reality distortion, value judgment distortion, and action distortion, with amplifying factors such as attachment and reliance/dependency [6]. In feedback-linked subsets, conversations rated as having moderate or severe disempowerment potential received higher rates of positive user feedback (thumbs-up) than baseline, and the prevalence of such potential increased over time. The authors emphasize that feedback-linked conversations are not representative, that feedback samples likely overrepresent extremes, that the study observes snapshots rather than longitudinal user belief, and that it cannot directly confirm distorted belief or harm. The defensible reading is that some potentially reality-distorting interactions are positively reinforced under current feedback designs, not that users were demonstrably misled.

This pattern is consistent with the broader sycophancy literature and with provider deployment notes [7,18]. Cheng and colleagues [8] recently reported across 11 contemporary models that AI systems affirm users more often than humans do, and that sycophantic responses reduce responsibility-taking and increase users’ conviction of their own correctness. Jain and colleagues [9] showed that deployment-time interaction context, including memory and personalization, increases agreement sycophancy and perspective mimesis. OpenAI’s public account of an April 2025 GPT-4o update documents that posttraining and feedback redesign can introduce sycophancy that was not present in the pretrained base model, and that the change was rolled back [18]. Together, these findings suggest that the necessary conditions for collusion-like dynamics arise from the interaction of preference-tuning and deployment design, not from pretraining alone.

## Existing Technical Mitigations

A balanced account requires acknowledgment that AI safety practice already addresses parts of this problem. At least five families of mitigation are active areas of work. Pretraining data curation includes quality filtering, deduplication, source mixing, and model-based selection [13,14]. Constitutional AI and reinforcement learning from AI feedback use explicit principles or AI-generated feedback to steer behavior beyond naive human preference labels [15]. Reward-model evaluation and ensembling are intended to detect and reduce overoptimization against imperfect proxies [16,17]. Grounded generation, retrieval augmentation, refusal and “I don’t know” training, and uncertainty calibration are increasingly used in high-stakes deployments [25]. Live monitoring and feedback redesign, illustrated by the disempowerment study and the GPT-4o rollback, allow providers to detect and respond to emergent sycophancy in production [6,18]. Open-problems analyses of reinforcement learning from human feedback emphasize that none of these methods is sufficient on its own and that defense in depth is required [26].

The narrower claim made here is that none of these mitigation families is yet organized around a clinically informed account of when user self-report is unreliable. Filter quality is not the same as clinical reliability. A constitution can encode helpfulness, harmlessness, and honesty without encoding reality-testing. Reward-model evaluation can audit calibration without auditing the clinical validity of the preferences being modeled. Grounding to authoritative documents does not address the unreliability of the user’s own first-person account. Red-teaming probes for jailbreaks and unsafe outputs but is not standardly resourced with psychiatric expertise in distorted cognition.

## Implications and Operationalized Proposals

### The Scope of the Problem

The implications extend beyond psychosis and beyond psychiatry. Any AI system that processes human input (a triage algorithm, a risk assessment tool, a diagnostic support system, or a patient-facing chatbot) operates under the assumption that user input broadly reflects clinical reality. The individual catastrophizing during a crisis, the person whose health-seeking behavior is anxiety-driven, and the patient whose account is shaped by years of institutionalization all generate input that current systems treat as ground truth.

International consensus guidelines for trustworthy AI in health care, including FUTURE-AI, articulate principles of fairness, universality, traceability, usability, robustness, and explainability and address data quality at a general level [27]. The narrower claim is that none of these frameworks specifically operationalizes *clinical reliability of self-report* as a data-quality dimension. World Health Organization guidance on large multimodal models [28], the EU AI Act’s high-risk classification for medical devices, the US Food and Drug Administration’s Good Machine Learning Practice, and the National Institute for Health and Care Excellence’s Evidence Standards Framework similarly do not yet address it.

Three first-pass proposals follow. They are programmatic and require empirical evaluation.

### Proposal 1: Clinical Input Into Preference Data, Red-Teaming, and Postmarket Surveillance

*Who:* AI developers, contracted clinical advisory groups, professional colleges (for example, the Royal College of Psychiatrists and the British Psychological Society in the United Kingdom; analogous bodies internationally). *Where in the life cycle:* preference labeling for assistants used in or affecting mental health contexts; red-team scenario design; postmarket live-conversation auditing. *How:* development of a clinical-reliability annotation schema for preference data, specifying when a candidate response should be preferred for challenging rather than affirming an apparently distorted account, with explicit attention to psychosis, mania, secondary gain, and crisis presentations. *Measurable output:* a published schema, interrater reliability statistics on a held-out set, and a comparison of model behavior on a clinical-reliability evaluation suite before and after schema-aligned preference training.

### Proposal 2: Routine Clinical Inquiry About AI Use

*Who:* psychiatric and clinical psychology services, undergraduate and postgraduate medical curricula, and curriculum bodies such as the Royal College of Psychiatrists. *Where:* psychosocial assessment, risk assessment, and Mental State Examination; safeguarding reviews; new-onset psychosis pathways. *How:* addition of explicit AI-use items to standard history-taking proformas, building on the AI-literacy competency proposal of Morrin and colleagues [4]. *Measurable output:* validated AI-use items in routine documentation; audit of capture rates; competency descriptors in core curricula.

### Proposal 3: Clinical Reliability of Training and Preference Data as a Trustworthy-AI Criterion

*Who:* trustworthy-AI framework authors (FUTURE-AI consortium, World Health Organization, regulators), with clinical input from psychiatry, clinical psychology, and lived-experience representation. *Where:* the data-quality and robustness sections of frameworks such as FUTURE-AI [27] and World Health Organization LLM guidance [28] and at the conformity-assessment stage for high-risk medical AI under the EU AI Act and equivalent regimes. *How:* an explicit criterion that, where systems will be deployed in mental-health–relevant contexts, training and preference data have been assessed for systematic biases that clinical practice identifies as undermining the reliability of self-report. *Measurable output:* a reliability-of-self-report data-quality item added to at least one international framework; audit checklists; reporting in deployment summaries.

## Limitations and Scope

This is a conceptual contribution, not an empirical study. Clinical examples, including mania, persecutory delusions, and severe depression, are illustrative rather than epidemiologically calibrated. The disempowerment study cited reports potential rather than confirmed distorted belief. Whether clinician involvement in data curation and preference labeling will improve downstream safety remains an empirical question, and any clinician-input scheme must itself be subject to governance: lived-experience representation, transparent criteria, and external audit are essential safeguards. The proposals are first-pass and require iterative empirical evaluation.

## Conclusions

The technical literature on AI alignment describes sycophancy, reward overoptimization, and disempowerment potential. The contribution offered here is a clinically informed redescription: collusion as an analytic frame, and the clinical reliability of training and preference data as a candidate criterion within trustworthy-AI guidance. Psychiatry and clinical psychology have developed standardized methods for assessing self-report reliability that are directly relevant to the design and governance of AI systems and are currently absent from those processes. Bringing this expertise into the pipeline will not solve sycophancy, but it may help name and address one dimension of the problem that purely technical mitigations are not yet organized to capture.

## Abbreviations

AI: artificial intelligence
LLM: large language model

## References

[1] Wang L, Bhanushali T, Huang Z, Yang J, Badami S, Hightow-Weidman L (2025). Evaluating generative AI in mental health: systematic review of capabilities and limitations. JMIR Ment Health.

[2] Clegg KA (2025). Shoggoths, sycophancy, psychosis, oh my: rethinking large language model use and safety. J Med Internet Res.

[3] Moore J, Grabb D, Agnew W (2025). Expressing stigma and inappropriate responses prevents llms from safely replacing mental health providers.

[4] Morrin H, Nicholls L, Levin M (2026). Artificial intelligence-associated delusions and large language models: risks, mechanisms of delusion co-creation, and safeguarding strategies. Lancet Psychiatry.

[5] Chung VHA, Bernier P, Hudon A (2026). Mass media narratives of psychiatric adverse events associated with generative AI chatbots: rapid scoping review. JMIR Ment Health.

[6] Sharma M, McCain M, Douglas R, Duvenaud D (2026). Who’s in charge? Disempowerment patterns in real-world LLM usage. arXiv.

[7] Sharma M, Tong M, Korbak T, Duvenaud D, Askell A, Bowman SR (2023). Towards understanding sycophancy in language models. arXiv.

[8] Cheng M, Lee C, Khadpe P, Yu S, Han D, Jurafsky D (2026). Sycophantic AI decreases prosocial intentions and promotes dependence. Science.

[9] Jain S, Park C, Viana M, Wilson A, Calacci D (2025). Interaction context often increases sycophancy in LLMs. arXiv.

[10] Rajkomar A, Hardt M, Howell MD, Corrado G, Chin MH (2018). Ensuring fairness in machine learning to advance health equity. Ann Intern Med.

[11] Birhane A, Isaac W, Prabhakaran V (2022). Power to the people? Opportunities and challenges for participatory AI.

[12] Reeves-McLaren N, Christensen SM (2025). Data integrity in materials science in the era of AI: balancing accelerated discovery with responsible science and innovation. J Mater Chem A.

[13] Li J, Fang A, Smyrnis G (2024). DataComp-LM: in search of the next generation of training sets for language models. arXiv.

[14] Albalak A, Elazar Y, Xie SM (2024). A survey on data selection for language models. Transactions on Machine Learning Research.

[15] Bai Y, Kadavath S, Kundu S, Askell A, Kernion J, Jones A (2022). Constitutional AI: harmlessness from AI feedback. arXiv.

[16] Coste T, Anwar U, Kirk R, Krueger D (2023). Reward model ensembles help mitigate overoptimization. arXiv.

[17] Frick E, Li T, Chen C (2024). How to evaluate reward models for RLHF. arXiv.

[18] (2025). Sycophancy in GPT-4o: what happened and what we’re doing about it. OpenAI.

[19] Tversky A, Kahneman D (1974). Judgment under uncertainty: heuristics and biases. Science.

[20] Kahneman D (2011). Thinking, Fast and Slow.

[21] Gigerenzer G, Brighton H (2009). Homo heuristicus: why biased minds make better inferences. Top Cogn Sci.

[22] Beck AT (1976). Cognitive Therapy and the Emotional Disorders.

[23] Palmier-Claus JE, Ainsworth J, Machin M (2012). The feasibility and validity of ambulatory self-report of psychotic symptoms using a smartphone software application. BMC Psychiatry.

[24] Saeb S, Zhang M, Karr CJ (2015). Mobile phone sensor correlates of depressive symptom severity in daily-life behavior: an exploratory study. J Med Internet Res.

[25] Kenthapadi K, Sameki M, Taly A (2024). Grounding and evaluation for large language models: practical challenges and lessons learned. arXiv.

[26] Casper S, Davies X, Shi C (2023). Open problems and fundamental limitations of reinforcement learning from human feedback. arXiv.

[27] Lekadir K, Frangi AF, Porras AR (2025). FUTURE-AI: international consensus guideline for trustworthy and deployable artificial intelligence in healthcare. BMJ.

[28] (2024). Ethics and governance of artificial intelligence for health: guidance on large multi-modal models. World Health Organization.

